# 

**Published:** 1862-07

**Authors:** 


					﻿New Series, Vol. 5, No. 7.
Whole Series, Vol. 19, No. 7.
THE CHICAGO
MEDICAL JOURNAL.
DANIEL BRAINARD, M. D.,
J. ADAMS ALLEN, M. D.,
Editors and Proprietors.
1862.
CHICAGO:
WM. PIOOTT, BOOK AND JOB PRINTER, 130 CLARK STREET.
1862.
				

